# Supplementary material for: Polymicrobial Biofilm Organization of Staphylococcus aureus and Pseudomonas aeruginosa in a Chronic Wound Environment
Source: Int J Mol Sci. 2022 Sep 15;23(18):10761. doi: 10.3390/ijms231810761 (PMC9504628; doi:10.3390/ijms231810761)
Supplement: Supplementary file 1 [file ijms-23-10761-s001.zip › ijms-1874308-supplementary.pdf]

Supplementary data

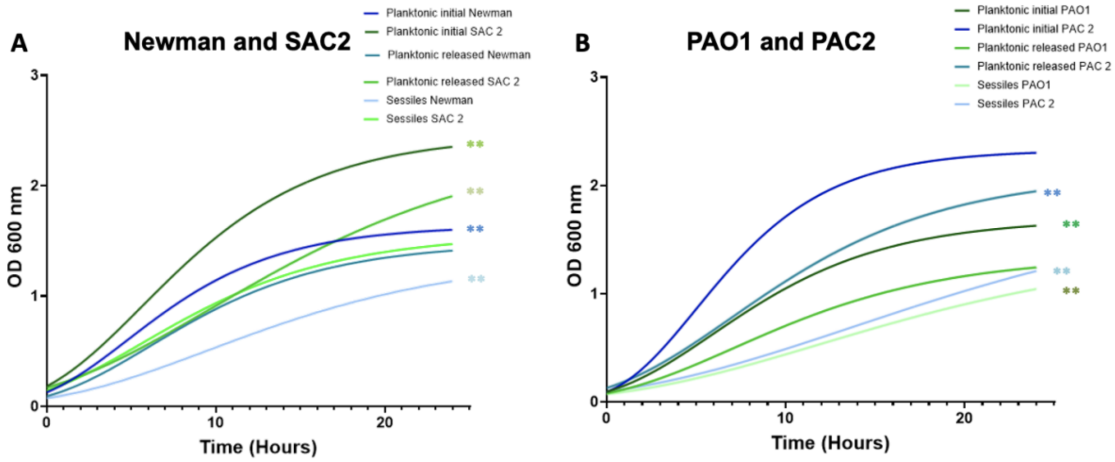

Figure S1. Growth curves of the reference strain Newman and clinical *S. aureus* strain SAC2 (A) and the reference strain PAO1 and clinical *P. aeruginosa* strain PAC2 (B) cultivated in CWM. Cultures were sampled at the indicated time points, measure of the OD600 were performed. Experiments were performed in three biological replicates. Results are presented as the mean ± standard deviation of three different experiments. Statistics were performed using a t-test on GraphPad Prism version 9.2 to compare planktonic released and sessile cells compared to initial bacteria. **p<0.01.

Table S1. Bacterial strains and media used in the study.

Strain	Characteristics	Reference	Antibiotic Resistance
PAO1	Reference strain	51	PIP
Newman	Reference strain	52	Wild type
SAC2	*S. aureus* ST15 clinical strain isolated on a DFI* (patient n°1)	This study	PCN; ERY
SAC4	*S. aureus* ST7 clinical strain isolated on a DFI (patient n°2)	This study	PCN; ERY
PAC2	*P. aeruginosa* clinical strain isolated on a DFI (patient n°1)	This study	PIP; ATM; CIP
PAC4	*P. aeruginosa* clinical strain isolated on a DFI (patient n°2)	This study	Wild type
**Media**			
BHI	Brain Heart Infusion, reference medium for bacterial culture	Sigma-Aldrich	
CWM	Chronic Wound Medium, medium mimicking *in vivo* conditions encountered in chronic wounds<br>**Composition :** 79,5 % Bolton broth vol/vol, 20% Heat-inactivated human serum vol/vol, 0,5% Hemolyzed human blood vol/vol, 1x10^6/ mL debris of human keratinocytes (HaCaT), NaOH 1M for a fixed pH to 8, 1% 1M HEPES	6	

*DFI, diabetic foot infection. PIP : Piperacillin, PCN : Penicillin G, ERY : Erythromycin, ATM : Aztreonam, CIP: Ciprofloxacin

Table S2. Primers used in the study.

Primers used and target function	Target region	Primer name	Oligonucleotide sequence	Reference
P. aeruginosa				
**Acyl homoserine lactone**	*lasI*	lasI-F<br>lasI-R	5′ – GCCCCTACATGCTGAAGAACA – 3′<br>5′- GTCCAGAGTTGATGGCGAAA – 3′	Aghamollaei, 2015
**Acyl homoserine lactone**	*rhII*	rhII-F<br>rhII-R	5′- AGCTTCTCGATGAAGACCTGATG -3′<br>5′- TGCTCTCTGAATCGCTGGAA - 3′	Mukherjee, 2017
**Exopolysaccharides**	*pel*	pel-F<br>pel-R	5′ – AGCAAGAAAGGAATCGCCG – 3′<br>5′ – GACCGACAGATAGGCGAAGG – 3′	Colvin, 2011
**Exopolysaccharides**	*psl*	psl-F<br>psl-R	5′- CTGCCCTCACCTTTCGCC – 3′<br>5′- GGAAGGATCAGCTGCG – 3′	Colvin, 2011

**Housekeeping gene**	*rpoD*	rpoD-F<br>rpoD-R	5′- CGATCGGTGACGACGAAGAT- 3′<br>5′- GTTCATGTCGATGCCGAAGC- 3′	Seder, 2021
S. aureus				
**α hemolysin**	*hla*	hla-F<br>hla-R	5′- TCCAGTGCAATTGGTAGTCA - 3′<br>5′- GGCTCTATGAAAGCAGCAGA - 3′	Otto, 2013
**Protein A**	*spa*	spa-F<br>spa-R	5′-TATGCCTAACTTAAATGCTG - 3′<br>5′- TTGGAGCTTGAGAGTCATTA- 3′	Otto, 2013
**Accessory gene regulator**	*agrA*	agrA-F<br>agrA-R	5′- CAAAGAGAAAACATGGTTACCATTATTAA -3′<br>5′- CTCAAGCACCTCATAAGGATTATCAG -3′	Garzoni, 2007
**MSCCRAM**	*fnbpA*	fnbpA-F<br>fnbpA-R	5′- AAATTGGGAGCAGCATCAGT - 3′<br>5′- GCAGCTGAATTCCCATTTTC - 3′	Pfaffl, 2001
**Housekeeping gene**	*gyrB*	gyrB-F<br>gyrB-R	5′- AGTAACGGATAACGGACGTGGTA - 3′<br>5′- CCAACACCATGTAAACCACCAGAT - 3′	Ngba Essebe, 2017

*MSCCRAM, microbial surface components recognizing adhesive matrix molecules

References

Aghamollaei, H.; Moghaddam, M.M.; Kooshki, H.; Heiat, M.; Mirnejad, R.; Barzi, N.S. Detection of *Pseudomonas aeruginosa* by a triplex polymerase chain reaction assay based on *lasI/R* and *gyrB* genes. *J Infect Public Health* **2015**, *8*, 314-322.

Colvin, K.M.; Gordon, V.D.; Murakami, K.; Borlee, B.R.; Wozniak, D.J.; Wong, G.C.; Parsek, M.R. The *pel* polysaccharide can serve a structural and protective role in the biofilm matrix of *Pseudomonas aeruginosa*. *PLoS Pathog* **2011**, *7*, e1001264.

Garzoni, C.; Francois, P.; Huyghe, A.; Couzinet, S.; Tapparel, C.; Charbonnier, Y.; Renzoni, A.; Lucchini, S.; Lew, D.P.; Vaudaux, P.; Kelley, W.L.; Schrenzel, J. A global view of *Staphylococcus aureus* whole genome expression upon internalization in human epithelial cells. *BMC Genomics* **2007**, *8*, 171.

Mukherjee, S.; Moustafa, D.; Smith, C.D.; Goldberg, J.B.; Bassler, B.L. The RhlR quorum-sensing receptor controls *Pseudomonas aeruginosa* pathogenesis and biofilm development independently of its canonical homoserine lactone autoinducer. *PLoS Pathog* **2017**, *13*, e1006504.

Ngba Essebe, C.; Visvikis, O.; Fines-Guyon, M.; Vergne, A.; Cattoir, V.; Lecoustumier, A.; Lemichez, E.; Sotto, A.; Lavigne, J.P.; Dunyach-Remy, C. Decrease of *Staphylococcus aureus* Virulence by *Helcococcus kunzii* in a *Caenorhabditis elegans* Model. *Front Cell Infect Microbiol* **2017**, *7*, 77.

Otto, M.P.; Martin, E.; Badiou, C.; Lebrun, S.; Bes, M.; Vandenesch, F.; Etienne, J.; Lina, G.; Dumitrescu, O. Effects of sub-inhibitory concentrations of antibiotics on virulence factor expression by community-acquired methicillin-resistant *Staphylococcus aureus*. *J Antimicrob Chemother* **2013**, *68*, 1524–1532.

Pfaffl, M.W., Hageleit, M. Validities of mRNA quantification using recombinant RNA and recombinant DNA external calibration curves in real-time RT-PCR. *Biotechnol Lett* **2001**, *23*, 275–282.

Seder, N.; Abu Bakar, M.H.; Abu Rayyan, W.S. Transcriptome Analysis of *Pseudomonas aeruginosa* Biofilm Following the Exposure to Malaysian Stingless Bee Honey. *Adv Appl Bioinform Chem* **2021**,*14*, 1-11.
